# Toxoplasmosis seroprevalence in Iranian women and risk factors of the disease: a systematic review and meta-analysis

**DOI:** 10.1186/s41182-017-0048-7

**Published:** 2017-04-12

**Authors:** Azadeh Mizani, Abbas Alipour, Mehdi Sharif, Shahabeddin Sarvi, Afsaneh Amouei, Azar Shokri, Mohammad-Taghi Rahimi, Seyed Abdollah Hosseini, Ahmad Daryani

**Affiliations:** 1 Toxoplasmosis Research Center, Mazandaran University of Medical Sciences, P. O. Box 48168-95475, Sari, Iran; 2 Parasitology and Mycology Department, School of Medicine, Mazandaran University of Medical Sciences, Sari, Iran; 3 Student Research Committee, Mazandaran University of Medical Sciences, Sari, Iran; 4 Community Medicine Department, School of Medicine, Mazandaran University of Medical Sciences, Sari, Iran

**Keywords:** Toxoplasmosis, *Toxoplasma gondii*, Pregnant women, Women, Girls, Seroprevalence, Systematic review, Iran

## Abstract

**Background:**

Toxoplasmosis is caused by an intracellular obligatory parasite, *Toxoplasma gondii*, and it has global distribution. The purposes of this systematic review and meta-analysis were to evaluate the seroprevalence of toxoplasmosis in Iranian pregnant women, and girls and women of childbearing age, and identify potentially preventable risk factors.

**Methods:**

Between November 2014 and February 2017, nine electronic databases that reported data on the *T. gondii* seroprevalence in Iranian women were searched. Our search resulted in 83 reports published from 1994 to 2017.

**Results:**

The results showed that the pooled estimation for the prevalence of *T. gondii* using a random-effect model was 43% (95% confidence interval (CI) = 38–48%) in pregnant women and 33% (95% CI = 23–43%) in girls and the childbearing age groups. There was a significant association between the *T. gondii* seroprevalence with age and the gestational age of conception in pregnant women and those who had contact with cats in both groups.

**Conclusions:**

This is the first comprehensive systematic review of *T. gondii* infection seroprevalence in Iranian women, which showed a high prevalence of *Toxoplasma* infection. Around 57% of pregnant women and 67% of girls and the childbearing age groups were seronegative and thus were susceptible to infection and should be monitored.

**Electronic supplementary material:**

The online version of this article (doi:10.1186/s41182-017-0048-7) contains supplementary material, which is available to authorized users.

## Background

Toxoplasmosis, a cosmopolitan infection in humans and animals, is caused by an intracellular obligatory parasite, *Toxoplasma gondii* [1]. *T. gondii* is spread worldwide, as at least one third of the world’s population is infected [2]. The broad geographic location of toxoplasmosis is associated with several risk factors such as the geographical climate, contact with cats or other pet’s faeces, nutritional habits, and location of one’s residence [3]. The worldwide prevalence of antibodies against *T. gondii* varies from 30 to 60% in both developing and developed countries [4]. Infection is acquired by accidentally ingesting oocysts in food, water, and soil contaminated with cat faeces or the consumption of raw meat containing tissue cysts [5, 6].

Toxoplasmosis can lead to life-threatening conditions in high-risk groups such as pregnant women, immunodeficient individuals (e.g. HIV-positive patients, organ transplant recipients, and patients with cancer) [7, 8]. Although *T. gondii* remains dormant in healthy individuals, exposure to parasites during pregnancy can lead to vertical transmission to the embryo [9]. The incidence of maternal infection during pregnancy is 1–8 per 1000 pregnancies [10]. A newborn exposed to *T. gondii* may develop congenital toxoplasmosis with microcephaly, hydrocephaly, blindness, spontaneous abortion, and stillbirth [9]. It may also significantly reduce the quality of life in children who survive prenatal infection and affect the socioeconomic burden on the patient’s family and the government [11]. It is important to determine whether infection occurs in the early stage of conception or before because women who have been exposed to infection prior to pregnancy are not at risk of having an infected child [12]. Exceptions have been seen in immunodeficient mothers [13]. Screening for anti-*Toxoplasma* antibodies in pregnant women and also non-immune girls of childbearing age is the mainstay for controlling and preventing congenital infection [9, 11, 14].

Because of its importance and the fact that *T. gondii* is ubiquitous, seroepidemiologic studies help establish the health policies of each country. The purposes of this systematic review and meta-analysis were as follows: (1) to evaluate the seroprevalence of toxoplasmosis in Iranian pregnant women, (2) to estimate the prevalence of *T. gondii* in girls and women of childbearing age, (3) to identify the potentially preventable risk factors most likely to have the greatest impact on the incidence of *Toxoplasma* infection in pregnancy, and (4) to determine whether seronegative women are a high-risk group for toxoplasmosis that need specific control and prevention strategies to reduce the level of *Toxoplasma* infection.

## Methods

The following four literature search strategies were used in this systematic review and meta-analysis: (1) computer search, (2) study selection, (3) data synthesis, and (4) data analysis and quality assessment.

### Computer search

Six English language databases (i.e. PubMed, ScienceDirect, Scopus, Google Scholar, ClinicalTrials.gov, and Cochrane Library) and three Persian databases (i.e. Scientific Information Database, Magiran, and Iran Medex) were searched from 1994 to 2017. The search was restricted to English- and Persian language databases. Medical Subject Heading terms and keywords included *Toxoplasma gondii*, toxoplasmosis, women, girl, pregnant women, childbearing age women, abortion, seroprevalence, seroepidemiology, Iran, and Islamic Republic of Iran. All citations were downloaded into EndNote.

### Study selection

Abstracts were reviewed independently by 4 authors, and cross-sectional studies that estimated the seroprevalence of *Toxoplasma* infection in pregnant women, women of childbearing age, women with an abortion, student girls from a university or high school, and girls referred to health centres for premarital laboratory tests or a medical laboratory for routine tests, except *T. gondii*, were selected for further use.

The final decisions about the inclusion or exclusion of studies were made separately after inspection. Discrepancies were resolved by discussion and consensus. In addition, the collected bibliographic publications were screened carefully, and duplicate articles, studies based in Iran, animal-based studies, and those with a specific population (e.g. the general population and all immunocompromised groups) were also excluded.

### Data synthesis

A protocol for data extraction was defined with groups. Data were extracted from selected publications using a data extraction sheet by authors, and then, the data were reviewed by a third author. The following data were extracted from the literature: first author, geographical region, the year of publication, diagnostic method with the cut-off value, total individuals, number of immunoglobulin (Ig)G-positive and IgG-negative cases, number of IgM-positive and IgM-negative cases, and number of both IgG- and IgM-positive cases among the study groups.

**Fig. 1:**
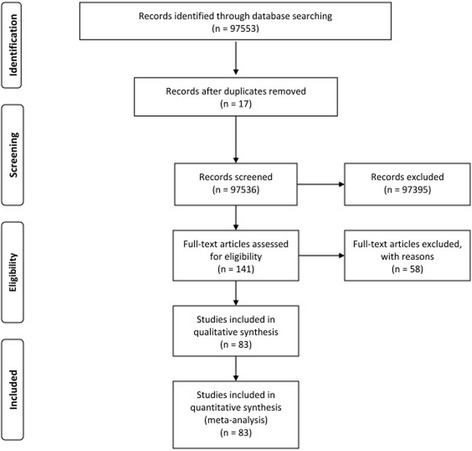
Flow diagram of the study design process

All information included demographic factors such as contact with cats and other animals, the consumption of raw fruits and vegetables, washing methods, the consumption of undercooked meat and unpasteurized milk, age, occupational group, education level, place of residence, number of pregnancies and abortions, and gestation age of the total individuals and positive cases. Whether the subjects wore gloves when cutting meat and had contact with soil was also collected. Figure 1 shows a diagram describing the study design process.

### Data analysis and quality assessment

The formula to calculate the population prevalence (weighed prevalence (WP)) of *T. gondii* in all groups or subgroups, included in this meta-analysis, was *P* = ∑ (pi) (1/vi)/∑ 1/vi. We used the metaprop command of STATA, version 12.1 (StataCorp, College Station, TX, USA), for pooling proportions. We estimated 95% confidence intervals (CIs) using the score statistic and the exact binomial method, and Freeman-Tukey double arcsine transformation of proportions via the ftt option. Forest plots were drawn to display the variation of the seroprevalence of the *Toxoplasma* infection test positivity rate among all studies together with the pooled measure and subgroup analysis. Heterogeneity of the prevalence estimates between studies was determined by the *Q* statistic and *I* ^2^ index, assuming that *I* ^2^ values of 25, 50, and 75% represented low, medium, and high heterogeneity, respectively. Therefore, we assess the quality of each study (Additional file 1: Table S1 and Additional file 2: Table S2). The influence of study characteristics (e.g. contact with cats and other pets; the consumption of raw meat, milk, soil, fruits, and vegetables; washing methods; age; occupational group; education level; place of residence; number of pregnancies and abortions; gestation age; the use of gloves when cutting meat; and study quality) was explored using subgroup analysis and meta-regression.

## Results

Of 97,553 literature searches from nine databases, 83 records were eligible to include in this systematic review and meta-analysis. Thirty-eight articles reported results about the seroprevalence of toxoplasmosis in women and girls of childbearing age, 45 about the seroprevalence of *Toxoplasma* infection among pregnant women. Approximately all the studies included in this meta-analysis had a cross-sectional design. Of 40,117 individuals, 16,698 cases had Ig against *T. gondii* and were included in the meta-analysis, followed by 39,837 individuals, with 15,822 positive cases for IgG, 16,590 with 652 positive cases for IgM, and 5447 with 224 cases positive for both IgG and IgM.

**Fig. 2:**
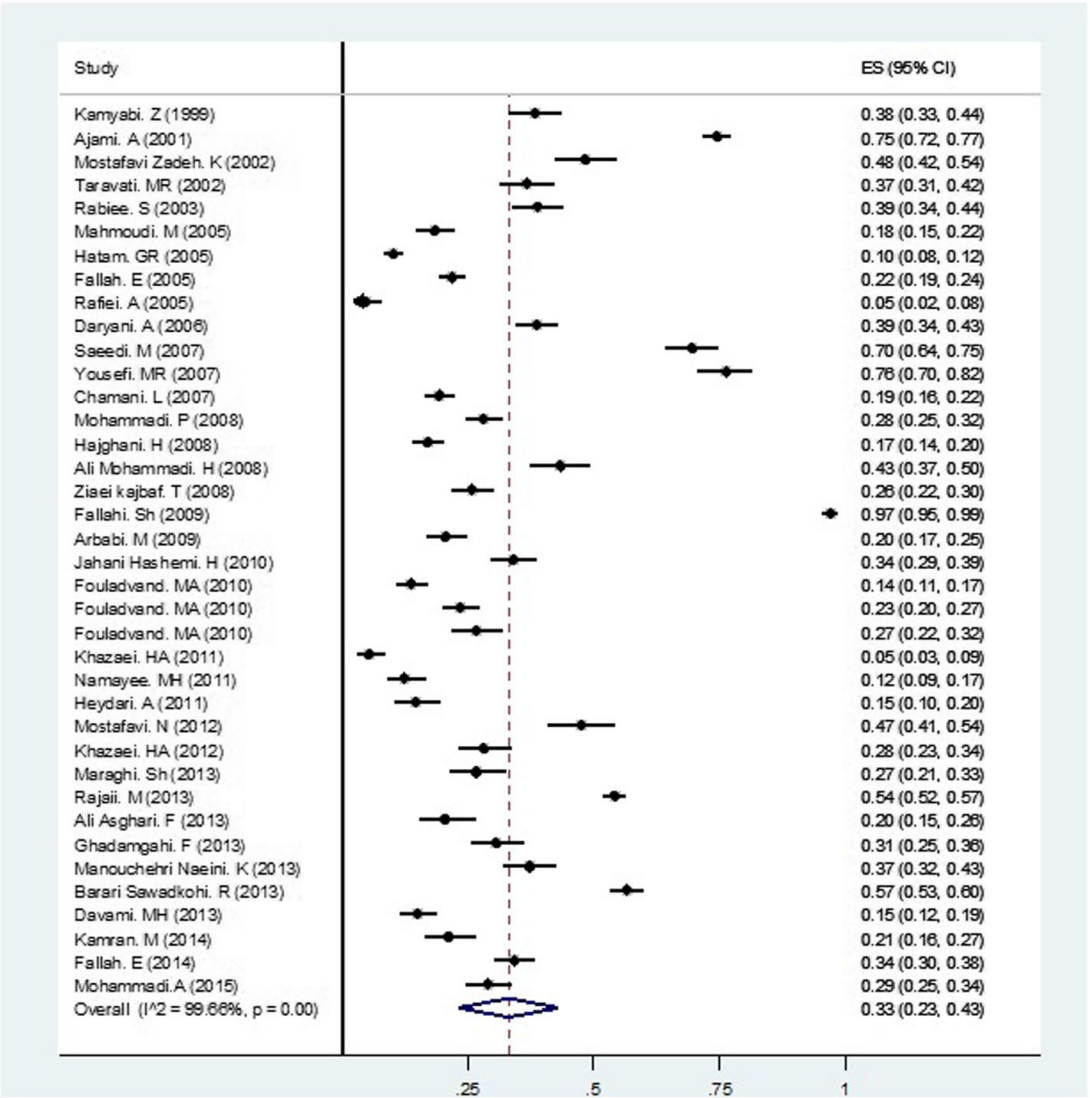
Random-effect meta-analysis of pooled estimation of *Toxoplasma gondii* in girls and women of childbearing age group

The prevalence of anti-*Toxoplasma* seropositivity in the girls group (i.e. women of childbearing age, high school girls, girl students from a university, girls referred to a health centre for pre-marriage examination, and non-pregnant women sent to a medical laboratory for examination) based on the random-effect model was 33% (95% CI = 23–43%). Figure 2 shows the forest plot diagram. About 67% of girls were seronegative and were not immune. The pooled estimations of IgG, IgM, and both IgG- and IgM-positive cases were 31% (95% CI = 21–41%), 5% (95% CI = 3–6%), and 3% (95% CI = 2–5%), respectively. Table 1 shows the baseline characteristic of the included studies.

**Table 1:** Girls and women of childbearing age group baseline characteristics of included studies

Province	Total individuals	IgG-positive cases (%)	IgM-positive cases (%)	IgG- and IgM-positive cases (%)	Total prevalence (%)	Authors
Kerman	350	103 (29.42)	18 (5.14)	13 (3.71)	134 (38.28)	Kamyabi et al., 1999
Mazandaran	980	731 (74.59)			731 (74.59)	Ajami et al., 2001
Isfahan	273	132 (48.35)			132 (48.35)	Mostafavi Zadeh et al., 2002
West Azerbaijan	300	98 (32.66)	12 (4)		110 (36.66)	Taravati et al., 2002
Hamedan	360	140 (38.88)			140 (38.88)	Rabiee et al., 2003
Isfahan	414	76 (18.35)			76 (18.35)	Mahmoudi et al., 2005
Fars	947	96 (10.13)			96 (10.13)	Hatamet al., 2005
East Azerbaijan	1000	218 (21.8)			218 (21.8)	Fallah et al., 2005
Khuzestan	259	12 (4.63)			12 (4.63)	Rafiei et al., 2005
Ardabil	504	175 (34.72)	20 (3.96)		195 (38.69)	Daryani et al., 2006
Golestan	300	145 (48.33)	35 (11.66)	29 (9.66)	209 (69.66)	Saeedi et al., 2007
Mazandaran	241	154 (63.9)	30 (12.44)		184 (76.34)	Yousefi et al., 2007
Tehran	707	119 (16.83)	17 (2.4)		136 (19.23)	Chamani et al., 2007
Kurdistan	600	169 (28.16)			169 (28.16)	Mohammadi et al., 2008
Kerman	549	93 (16.93)			93 (16.93)	Haj ghani et al., 2008
Ardabil	272	115 (42.27)		3(1.1)	118 (43.38)	Ali Mohammadi et al., 2008
Khuzestan	400	103 (25.75)			103 (25.75)	Ziaei kajbaf et al., 2008
Lorestan	465	452 (97.2)			452 (97.2)	Fallahi et al., 2009
Isfahan	400	79 (19.75)	3 (0.75)		82 (20.5)	Arbabi et al., 2009
Qazvin	400	136 (34)			136 (34)	Jahani Hashemi et al., 2010
Bushehr	491	54 (10.99)	8 (1.62)	5(1.01)	67 (13.64)	Fouladvand et al., 2010
Bushehr	516	114 (22.09)	7 (1.35)		121 (23.44)	Fouladvand et al., 2010
Bushehr	303	71 (23.43)	10 (3.3)		81 (26.73)	Fouladvand et al., 2010
Sistan and Baluchestan	280		15 (5.35)		15 (5.35)	Khazaie et al., 2011
South Khorasan	300	37 (12.33)			37 (12.33)	Namayee et al., 2011
Razavi Khorasan	240	35 (14.58)			35 (14.58)	Heydari et al., 2011
Isfahan	217	103 (47.46)			103 (47.46)	Mostafavi et al., 2012
Sistan and Baluchestan	280	79 (28.21)			79 (28.21)	Khazaei et al., 2012
Khuzestan	240	28 (11.66)	29 (12.08)	7 (2.91)	64 (26.66)	Maraghi et al., 2013
East Azerbaijan	1659	898 (54.12)			898 (54.12)	Rajaii et al., 2013
North Khorasan	215	44 (20.46)			44 (20.46)	Ali Asghari, et al., 2013
Tehran	300	85 (28.33)	4 (1.33)	3 (1)	92 (30.66)	Ghadamgahi et al., 2013
Mazandaran	800	453 (56.62)			453 (56.62)	Barari Sawadkohi et al., 2013
Fars	403	52 (12.9)	8 (1.98)		60 (14.88)	Davami et al., 2013
Chaharmahal and Bakhtiari	338	126 (37.27)			126 (37.27)	Manouchehri et al., 2014
Ilam	260	55 (21.15)			55 (21.15)	Kamran et al., 2014
East Azerbaijan	549	88 (16.02)	66 (12.02)	34 (6.19)	188 (34.24)	Fallah et al., 2014
Markazi	400	97 (24.25)	19 (4.75)		116 (29)	Mohammadi et al., 2015

Data analysis showed that there was a significant relationship between *Toxoplasma* seropositivity and contact with cats (*P* = 0.04). Although the seroprevalence of *Toxoplasma* infection was higher among individuals who had direct contact with cats (30%); groups without contact with other animals (26%); individuals who consumed raw meat (34%), milk (45%), and fruits or vegetables (24%); individuals who washed vegetables or fruits with water (27%) and cut meat without wearing gloves (18%); individuals who had a low education level (63%); and those who were employed (53%) and were aged >20 years, there were no associations between these aforementioned factors and the rate of *Toxoplasma* infection. Table 2 shows the overall characteristics of the risk factors of the included studies.

**Table 2:** Risk factors associated to seropositivity for *Toxoplasma gondii* in girls and women of childbearing age group

Demographic factors	Total individuals	Positive cases	Pooled estimation (%)	*I* ^2^	*P* value^a^
Age					0.62
≤20 years	2296	1040	45 (95% CI = 30 to 60)	98.91	
>20 years	4370	2294	39 (95% CI = 23 to 56)	98.49	
Contact with cat					0.04
Yes	1775	474	30 (95% CI = 24 to 37)	87.44	
No	3898	815	21 (95% CI = 16 to 26)	93.78	
Contact with other animals					0.41
Yes	657	182	26 (95% CI = 18 to 35)	82.33	
No	1443	295	21 (95% CI = 12 to 30)	94.98	
Contact to meat					0.98
Yes	646	204	28 (95% CI = 16 to 42)	89.59	
No	339	82	23 (95% CI = 19 to 28)	96.53	
Meat consumption					0.12
Undercooked	1755	466	34 (95% CI = 23 to 46)	95.33	
Cooked	2209	381	17 (95% CI = 12 to 22)	89.25	
Raw vegetable/fruit consumption					0.25
Yes	2645	631	24 (95% CI = 17 to 31)	94.49	
No		47	17 (95% CI = 9 to 27)	71.32	
Milk consumption					0.17
Pasteurized	1264	276	21 (95% CI = 8 to 38)	97.87	
Unpasteurized	95	34	45 (95% CI = 19 to 72)	78.93	
Educational status					0.37
Illiterate	211	114	63 (95% CI = 37 to 86)	91.70	
Junior/senior	1064	553	50 (95% CI = 30 to 70)	97.73	
High school	987	555	52 (95% CI = 24 to 80)	98.89	
Diploma/university	863	320	47 (95% CI = 18 to 77)	98.80	
Occupational group					0.37
Employee	572	340	53 (95% CI = 33 to 74)	99.17	
Housewife	2389	1336	52 (95% CI = 30 to 73)	95.28	
Students	418	133	83 (95% CI = 16 to 63)	95.95	
Wearing gloves during cutting meat					0.47
Yes	250	31	12 (95% CI = 8 to 17)	96.78	
No	756	137	18 (95% CI = 15 to 20)	96.78	
Washing vegetable/fruit					0.65
Water	573	176	27 (95% CI = 12 to 46)	95.36	
Antiseptic	705	107	19 (95% CI = 10 to 31)	89.76	
Residence					0.99
Urban	5137	2011	33 (95% CI = 19 to 49)	99.27	
Rural	2924	1213	33 (95% CI = 22 to 46)	97.53	

^a^Meta-regression analysis

**Fig. 3:**
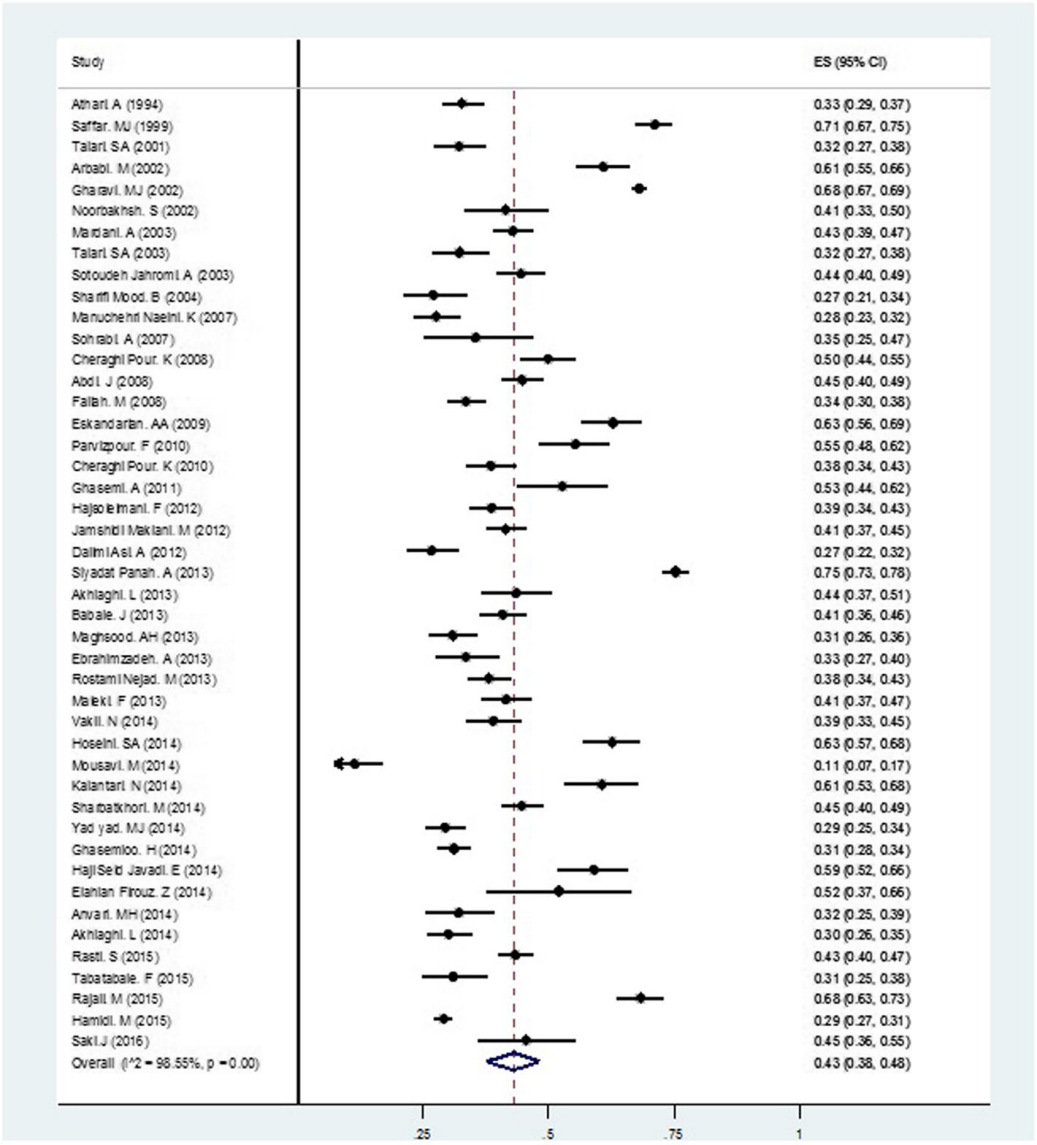
Forest plot diagram of total prevalence of *Toxoplasma gondii* in pregnant women group

The pooled estimation for the prevalence of anti-*Toxoplasma* antibodies in pregnant women using the random-effect model was 43% (95% CI = 38–48%). Figure 3 shows the forest plot diagram. The data indicated that 57% of pregnant women were seronegative and were not immune during pregnancy. Table 3 demonstrates the baseline characteristics of the included data. The pooled estimates of IgG, IgM, and both IgG- and IgM-positive cases were 40% (95% CI = 35–45%), 3% (95% CI = 2–4%), and 4% (95% CI = 2–5%), respectively.

**Table 3:** Pregnant women group baseline characteristics of included studies

Province	Total individuals	IgG-positive cases (%)	IgM-positive cases (%)	IgG- and IgM-positive cases (%)	Total prevalence (%)	Authors
Kermanshah	495	162(32.72)			162(32.72)	Athari et al., 1994
Mazandaran	612	435(71.07)			435(71.07)	Saffar et al., 1999
Isfahan	317	98(30.91)	4(1.26)		102(32.17)	Talari et al., 2001
Isfahan	340	207(60.88)			207(60.88)	Arbabi et al., 2002
Tehran	4120	2804(68.05)			2804(68.05)	Gharavi et al., 2002
Tehran	140	48(34.28)	10(7.14)		58(41.42)	Noorbakhsh et al., 2002
Isfahan	270	72(26.66)	15(5.55)		87(32.22)	Talari et al., 2003
Hormozgan	418	143(34.21)	33(7.89)	10(2.39)	186(44.49)	SotoudehJahromi et al., 2003
Qom	600	257(42.83)			257(42.83)	Mardani et al., 2003
Sistan and Baluchestan	200	54(27)			54(27)	Sharifi Mood et al., 2004
Chaharmahal and Bakhtiari	384	106(27.60)			106(27.60)	Manuchehri et al., 2007
Khuzestan	79	28(35.44)			28(35.44)	Sohrabi et al., 2007
Ilam	553	247(44.66)			247(44.66)	Abdi et al., 2008
Hamedan	576	193(33.50)			193(33.50)	Fallah et al., 2008
Qazvin	255	160(62.74)			160(62.74)	Eskandarian et al., 2009
Kurdistan	201	54(26.86)	24(11.94)	33(16.41)	111(55.22)	Parvizpour et al., 2010
Lorestan	390	121(31.02)	29(7.43)		150(38.46)	Cheraghi Pour et al., 2010
Lorestan	331	130(39.27)	35(10.57)		165(49.84)	Cheraghi Pour et al., 2010
Isfahan	127	67(52.75)			67(52.75)	Ghasemi et al., 2011
Zanjan	500	186(37.2)	7(1.4)		193(38.60)	Hajsoleimani et al., 2012
Hormozgan	608	252(41.44)			252(41.44)	JamshidiMakiani et al., 2012
East Azerbaijan	300	79(26.33)	1(0.33)		80(26.66)	DalimiAsl et al., 2012
Mazandaran	1057	739(69.91)	57(5.39)		796(75.30)	SiyadatPanah et al., 2013
Qom	200	76(38)	11(5.5)		87(43.50)	Akhlaghi et al., 2013
Razavi Khorasan	419	144(34.36)		27(18.75)^a^	171(40.81)	Babaie et al., 2013
Hamedan	350	105(30)		3(2.85)^b^	108(30.85)	Maghsood et al., 2013
Sistan and Baluchestan	221	68(30.76)	3(1.35)	3(1.35)	74(33.48)	Ebrahimzadeh et al., 2013
Lorestan	496	154(31.04)		35(22.72)^c^	189(38.10)	RostamiNejad et al., 2013
Qom	400	145(36.25)	21(5.25)		166(41.50)	Maleki et al., 2013
Markazi	308	117(37.98)		3(1.74)^d^	120(38.96)	Vakil et al., 2014
Mazandaran	289	170(58.82)	7(2.42)	4(1.38)	181(62.62)	Hoseini et al., 2014
Sistan and Baluchestan	185	19(10.27)	1(0.54)	1(0.54)	21(11.35)	Mousavi et al., 2014
Mazandaran	175	106(60.57)			106(60.57)	Kalantari et al., 2014
Golestan	555	221(39.81)	19(3.42)	8(1.44)	248(44.68)	Sharbatkhori et al., 2014
Khuzestan	501	137(27.34)	7(1.39)	3(0.59)	147(29.34)	Yadyad et al., 2014
Tehran	785	244(31.08)			244(31.08)	Ghasemloo et al., 2014
East Azerbaijan	195	115(58.97)			115(58.97)	Haji SeidJavadi et al., 2014
Mazandaran	50	26(52)			26(52)	ElahianFirouz et al., 2014
Yazd	181	58(32.04)			58(32.04)	Anvari et al., 2014
Alborz	400	116(29)	4(1)		120(30)	Akhlaghi et al., 2014
Isfahan	798	341(42.73)	5(0.62)		346(43.35)	Rasti et al., 2015
Qazvin	200	58(29)	4(2)		62(31)	Tabatabaieet al., 2015
East Azerbaijan	391	267(68.28)			267(68.28)	Rajaii et al., 2015
Hamedan	2523	681(26.99)	51(2.02)		732(29.01)	Hamidi et al., 2015
Khuzestan	110	47(42.72)	3(2.72)		50(45.45)	Saki et al., 2016

^a^Total individuals for IgM seropositivity is 144

^b^Total individuals for IgM seropositivity is 105

^c^Total individuals for IgM seropositivity is 154

^d^Total individuals for IgM seropositivity is 172

In this group, there was a significant association among age groups (*P* = 0.004), contact with cats (*P* = 0.005), gestation age of the pregnant women (*P* = 0.001), and the rate of infection. Data indicated that although the prevalence of *T. gondii* infection was higher among individuals who had direct contact with soil (42%); consumed raw meat (41%) and fruits or vegetables (39%); washed vegetables or fruits with water (39%); lived in rural areas (47%); had a senior-junior high school education level (39%); and were aged >30 years (53%), there were no associations between these aforementioned factors and *T. gondii* antibody positivity. Table 4 shows the overall characteristics of the described risk factors.

**Table 4:** Risk factors associated to seropositivity for *Toxoplasma gondii* in pregnant women group

Demographic factors	Total individuals	Positive cases	Pooled estimation (%)	*I* ^2^	*P* value^a^
Age					0.004
≤30 years	5332	2041	34 (95% CI = 26 to 43)	97.69	
>30 years	1414	772	53 (95% CI = 45 to 61)	87.70	
Contact with cat					0.005
Yes	1779	787	44 (95% CI = 36 to 52)	89.17	
No	2410	716	27 (95% CI = 20 to 34)	92.53	
Contact with soil					0.92
Yes	63	27	42 (95% CI = 30 to 54)	92.72	
No	929	310	33 (95% CI = 30 to 36)	92.72	
Meat consumption					0.4
Undercooked	1450	603	41 (95% CI = 28 to 53)	95.05	
Cooked	1814	645	35 (95% CI = 29 to 42)	88.62	
Raw vegetable/fruit consumption					0.46
Yes	1463	570	39 (95% CI = 25 to 54)	96.90	
No	520	164	31 (95% CI = 26 to 36)	29.12	
Washing vegetable/fruit					0.64
Water	847	358	39 (95% CI = 21 to 58)	96.85	
Antiseptic	602	212	34 (95% CI = 23 to 47)	88.09	
Residence					0.55
Rural	1796	762	47 (95% CI = 38 to 55)	91.72	
Urban	2361	1017	43 (95% CI = 34 to 52)	94.57	
Occupational group					0.94
Employee	143	68	47 (95% CI = 35 to 59)	48.56	
Housewife	1359	594	47 (95% CI = 39 to 55)	86.58	
Educational status					0.67
Illiterate	329	108	34 (95% CI = 25 to 44)	63.80	
Junior/senior	1617	665	39 (95% CI = 32 to 47)	88.35	
High school/diploma	1410	457	31 (95% CI = 26 to 36)	73.55	
University	493	189	36 (95% CI = 27 to 45)	76.63	
Gestation age					0.001
1st trimester	570	258	46 (95% CI = 40 to 51)	46.05	
2nd trimester	491	192	39 (95% CI = 35 to 43)	0	
3rd trimester	594	193	32 (95% CI = 29 to 36)	0	
Number of pregnancy					0.42
Null gravid	363	143	41 (95% CI = 23 to 61)	92.76	
Multi-gravid	452	228	53 (95% CI = 36 to 69)	92.16	
History of abortion					0.22
Yes	4285	1666	44 (95% CI = 37 to 50)	94.60	
No	3058	1170	38 (95% CI = 32 to 44)	90.93	

^a^Meta-regression analysis

## Discussion

*T. gondii* has a wide range of distribution, and seroprevalence data indicate that toxoplasmosis is one of the most common human infections worldwide [15]. Acute infection with *Toxoplasma* during pregnancy and its outcome for the foetus and newborn continue to occur in Iran and in other countries, despite the fact that it can be prevented. A large body of the research about the seroepidemiology of toxoplasmosis in women has been performed in Iran because of the great importance of *T. gondii* as a public health problem, but there is a lack of comprehensive systematic and documented data in this area.

Among all nine databases searched from November 2014 to February 2017, 83 records involving 40,117 individuals and 16,698 cases containing immunoglobulin against *T. gondii* in groups of women were eligible to be included in this systematic review and meta-analysis. The present study showed that the seroprevalence of anti-*T. gondii* antibodies in Iranian pregnant women and women of childbearing age were 43 and 33%, respectively. A higher prevalence of anti-*T. gondii* antibodies was observed in previous studies conducted in Iran: 55% in patients who had received transplants, 50% in HIV-infected individuals, and 45% in patients with cancer [16]. The prevalence of toxoplasmosis from a survey administered to the general population in Iran was 39.3% [9]. The data indicated that the seroprevalence of *T. gondii* in pregnant women in Europe varies from 9 to 67% [17]. Although some Asian countries such as Malaysia, India, and Nepal have the highest seroprevalence of *Toxoplasma* infection in pregnant women (41.8–55.4%), conversely, Korea and Vietnam report a low seropositivity rate of *T. gondii* (0.8 and 11.2%, respectively) [18–20]. In our neighbouring countries, Turkey and Pakistan, the anti-*Toxoplasma* antibody in pregnant women was found to be 33 and 19.25%, respectively [21, 22]. However, in Turkey, the seroprevalence of *Toxoplasma* infection in women of reproductive age was determined to be 58.3 and 1% for IgG and IgM, respectively [23].

*Toxoplasma* infection’s geographical distribution is related to several environmental factors (i.e. food habits, variations in climate, and contact with infected cat faeces) and sociodemographic factors (i.e. age, occupation, education, and residence) [24]. Any of these factors alone has little effect on the epidemiological status of *Toxoplasma* infection, but together, they can change the distribution pattern of the disease worldwide [9].

The geographical climate is one of the main factors of the disease. Our country has an arid climate, but the weather condition is variable. Some provinces such as Tehran, Fars, Kerman, and Khuzestan have various climates. Thus, an epidemiologic survey in this part of Iran is more difficult to administer. This variation of climatic conditions can introduce biases into the prevalence rate of *Toxoplasma* infection. In the northern part of Iran (around the Caspian Sea), there is a high prevalence of *T. gondii* infection, whereas in cold mountainous and hot provinces, lower seropositivity rates are found. The right climate status in the northern part provides a suitable condition for oocyst sporulation and survival in the environment. In mild and wet climates, the maturation of oocysts and transmission to a new host happens [9, 25]. Additional file 3: Figure S1 shows the prevalence of total *Toxoplasma* antibodies in both girls and pregnant women in all parts of Iran.

The abundance of cats is another risk factor of *Toxoplasma* infection. Domestic cats are one of the main sources of infection. Our results showed that the anti-*Toxoplasma* antibody in cat and other pet owners was higher than that in individuals who did not own or have contact with domestic animals in both pregnant girls and those of childbearing age. Similar findings were reported by other studies [26, 27]. Cats excrete millions of oocysts after ingestion of even one tissue cyst or bradyzoite. In the environment, sporulated oocysts can outlast for months or even years in moist soil. Terrestrial insects such as earthworms, beetles, and even flies can extend oocysts from the soil, which leads to food contamination. Cats may be infected when hunting small mammals contaminated by tissue cysts. They can shed a large amount of oocysts via faeces in the environment. Food, water, farms, and gardens may be contaminated by their oocysts. Keeping cats in the house (e.g. keeping them indoors or outdoors), especially in high-risk groups such as seronegative pregnant women who are susceptible to *Toxoplasma* infection, needs more consideration [28]. Previous data have indicated that dog fur that has come in contact with cat faeces may be one of the factors of oocyst transmission to humans [2].

In our study, *T. gondii* showed a higher antibody titre in individuals who consume undercooked meat, milk, vegetables, and fruits and washed them with water only instead of detergent in both aforementioned groups. Lopes et al. and Gelaye et al. showed the same results [26, 29]. The consumption of meat from domestic animals contaminated with tissue cysts is one of the main sources of infection. The risk of acquiring *T. gondii* via contaminated meat with tissue cysts varies between cultural and eating habits in various communities. However, meat is one of the major sources of Iranian meals; thus, the consumption of undercooked meat can lead to the transmission of parasites. There is a risk of *Toxoplasma* infection by consuming dairy products. Tachyzoites are orally infectious because they can survive in pepsin and trypsin. The consumption of milk containing tachyzoites in girls and women of childbearing age with no prior contact to the organism can lead to horizontal transmission and clinical infection. In seronegative women during pregnancy, the consumption of undercooked meats such as kebab (a meat product like sausage), dairy products, raw vegetables, and fruits can lead to congenital toxoplasmosis, which is a serious problem [9, 30].

Our data showed that the seropositivity of *T. gondii* infection among various occupations in both groups was different. In women of childbearing age and the girls group, the seroepidemiology of *T. gondii* in employees was a little higher than other jobs, but this difference was not significant. Our result was comparable to a study performed in Brazil, which showed a high prevalence of infection in this group [27]. However, in pregnant women, antibody titres were the same in both groups. Women spend more time cooking, tasting foods during meal preparation, handling and chopping meat in the kitchen, taking care of pets at home, gardening, and cleaning and washing vegetables and fruits. This finding explains why the seropositivity of infection in individuals who have contact with soil during gardening and women who chop meat without wearing gloves is higher than that in groups who do not have exposure to soil and raw meat. In various parts of the world, *T. gondii* oocysts have been isolated from soil samples [2].

Published studies have indicated that in women of childbearing age, by increasing the level of education, a rising trend in *T. gondii* seropositivity was observed. Evidence from a study by Hung et al. supported our finding [31]. The authors suggested that women with an academic education should have more knowledge related to *Toxoplasma* biology and its prevention and control strategies. The lack of effective information about this disease such as the route of transmission during pregnancy and poor socioeconomic status can increase the risk of infection.

The seroprevalence and serologically positive cases increased by age in pregnant groups. Nimiri et al. demonstrated that a higher seropositivity rate was seen in older age groups than in younger age groups [32]. The reason for this has not yet been discussed and it is not clear, but authors suggested the following reasons for infection: the prolonged exposure to the risk factors, transmission route, and lack of public awareness about preventive methods.

In the current study, the seroprevalence of *T. gondii* in pregnant women who lived in rural areas was higher than those in urban areas. In 2009, Lopes et al. found similar results with our findings [26]. During the first trimester of pregnancy, the risk of acquired congenital infection and impact on the foetus is 10–15%. However, in the second and third trimesters, the risk of foetal infection increases (60–90%) and also the effects on the foetus are milder [30, 33].

In the present study, the seropositivity rate of infection in the first trimester of pregnancy was higher than that in the second and third trimesters. In all women during conception, ideally in the first trimester, a serological screening test for *T. gondii* IgG and IgM detection are feasible [34]. This kind of screening helps clinicians detect seroconversion individuals and provide early treatment for infection [35]. In our study, multi-gravid women had a higher prevalence of *T. gondii* infection, and the prevalence was proportionally increased with an increasing number of children. Null gravid women had a prevalence of 41%, whereas women with more than one child had a prevalence of 53%. Our results showed a trend towards an increasing risk of positive *T. gondii* antibodies with increasing parity. In our study, the rate of anti-*Toxoplasma* antibodies among women with spontaneous abortion was 44%. This finding is in agreement with reports from Taiwan and Ethiopia [29, 31].

The most commonly used serological method of available data on *T. gondii* epidemiologic survey in both groups was ELISA. ELISA is a quantitative and inexpensive tool with a sensitivity and specificity of 100 and 98.4%, respectively [24]. The second common serological method is IFA (sensitivity, 95%; specificity, 96%). Albeit IFA is cheap, manageable, and safe, it is a manual tool and ultraviolet light microscopy is needed [9]. Additional file 4: Table S3 and Additional file 5: Table S4 summarize the characteristic of methods used in the included studies.

The prevention and control of *Toxoplasma* infection rely on sufficient information about parasite epidemiology. The knowledge on *T. gondii* seroprevalence, risk factors, and prevention strategies can lead to efficient incidence reduction [36]. The general population knows little about congenital toxoplasmosis and the risk of disease. Awareness of the risk factors of toxoplasmosis makes it possible for health authorities to define specific preventive strategies for high-risk populations. Primary prevention includes educational materials that contain the routes of how to prevent infection in women during pregnancy, which have resulted in reducing the prevalence of the seroconversion population.

Secondary prevention includes serological screening. The transmission of *T. gondii* to the foetus always happens during or before conception. In healthy women with previous infection, transmission to the foetus rarely occurs [37]. In both IgG- and IgM-negative cases, there is no serologic evidence of prior exposure to *T. gondii*, and the risk of congenital toxoplasmosis is high. Thus, they require serial testing during gestation. Anti-*Toxoplasma* IgG-positive women should be tested for the IgM antibody, and in IgM-positive cases, confirmative tests are needed. In women with IgG-positive and IgM-negative antibodies, the gestation age is very important. In women at <18 weeks of gestation, it means past infection and the risk of congenital toxoplasmosis is zero. However, in women at >18 weeks of gestation, it is difficult to predict whether exposure happened in the past or during conception. In anti-*Toxoplasma* IgG-negative and IgM-positive cases, a serological test should be repeated after 3 weeks. In this group, two conditions can occur: the antibody titre persists (IgG− and IgM+) or the IgG antibody changes to positive (IgG+ and IgM+). Management of the first group is the same as that for IgG− and IgM− patients, and in the second group, seroconversion occurs. In this group, the risk of *T. gondii* transmission to the foetus is high, and treatment, amniotic fluid polymerase chain reaction, and ultrasound should be performed [34].

The results show that there is heterogeneity across the studies as illustrated by the forest plot. The dissimilarity, sensitivity, and specificity of several methods used in various studies, differences in the sample size, nutritional habits, and environmental factors such as the geographical climate can result in this heterogeneity.

There are many studies based on the seroepidemiologic survey on women with toxoplasmosis in Iran, but there has not been a comprehensive systematic and documented report on this topic, except two articles on the epidemiology of *Toxoplasma* in women, which were not comprehensive [10, 38]. The present article fulfils this gap. Our systematic review and meta-analysis has certain limitations. The major limitations include the following: (1) there was no uniform sample size, (2) different methods with various sensitivity and specificity were used, (3) and the epidemiological results were heterogeneous. These aforementioned factors may have biased the prevalence of *Toxoplasma* infection in the population of Iranian women.

## Conclusions

This is the first comprehensive systematic review and meta-analysis of *T. gondii* in the overall population of women in our country. The data showed that 57% of Iranian pregnant women and 67% of girls and women of childbearing age were seronegative for *Toxoplasma* and susceptible to acute infection. Seronegative women are a high-risk group for congenital toxoplasmosis and should be monitored. We suggest that peer-led educational activities such as seminars and workshops are very effective, but they require great organizational efforts and lots of money and time. In Iran, there is no screening programme for women or seronegative pregnant women during gestation, but spouses are referred to health centres for premarital laboratory tests. It is necessary for the Ministry of Health to establish a screening programme and training course for couples who are referred to counselling departments for pre-marriage examinations. The risk factors for *Toxoplasma* should be documented in pamphlets and brochures with illustrations to make them easy to understand. However, didactic videos may serve as an effective teaching tool in these places. Importantly, health care organizations need to implement vast seroepidemiologic surveys on *Toxoplasma* infection in women and the disease risk factors using standardized methods, an identical sample size, and unique risk factors, to determine the true seropositivity rate in all provinces.

## Additional files

Additional file 1: Table S1. Girls group quality assessment table. (DOC 81 kb)

Additional file 2: Table S2. Pregnant women group quality assessment table. (DOC 92 kb)

Additional file 3: Figure S1. Prevalence rate of *Toxoplasma* total antibodies in both groups in different provinces of Iran. (JPG 380 kb)

Additional file 4: Table S3. Girls and women of childbearing age group methods of included data. (DOCX 16 kb)

Additional file 5: Table S4. Pregnant women group methods of included data. (DOCX 16 kb)
