# Supplementary material for: Toxoplasmosis seroprevalence in Iranian women and risk factors of the disease: a systematic review and meta-analysis
Source: Trop Med Health. 2017 Apr 12;45:7. doi: 10.1186/s41182-017-0048-7 (PMC5389165; doi:10.1186/s41182-017-0048-7)
Supplement: Supplementary file 1 — Girls group quality assessment table. (DOC 81 kb) [file 41182_2017_48_MOESM1_ESM.doc]

**Girls Group Quality Assessment Table**

| **Score** | **appropriate statistical methods**  **8** | **valid of survey measures/instruments**  **7** | **reliable of survey measures/instruments**  **6** | **standardized data collection methods**  **5** | **adequate response rate**  **4** | **sample characteristics matching the overall population**  **3** | **representativeness of probability sampling2** | **clear definition of the target population 1** | **Year** | **Author** | **No** |
| --- | --- | --- | --- | --- | --- | --- | --- | --- | --- | --- | --- |
| 6 | + | + | + | + | - | - | + | + | 1999 | Kamyabi. Z | 1 |
| 5 | - | + | + | + | - | - | + | + | 2001 | Ajami. A | 2 |
| 4 | + | - | - | + | - | + | - | + | 2002 | Mostafavi Zadeh. K | 3 |
| 4 | - | + | + | + | - | - | - | + | 2002 | Taravati. MR | 4 |
| 5 | + | + | + | + | - | - | + | - | 2003 | Rabiee. S | 5 |
| 6 | + | + | + | + | - | - | + | + | 2005 | Mahmoudi. M | 6 |
| 5 | + | + | + | + | - | - | - | + | 2005 | Hatam. GR | 7 |
| 6 | + | + | + | + | - | - | + | + | 2005 | Fallah. E | 8 |
| 6 | + | **+** | **+** | + | - | - | + | + | 2005 | Rafiei. A | 9 |
| 5 | + | + | + | + | - | - | - | + | 2006 | Daryani. A | 10 |
| 6 | + | + | + | + | - | - | + | + | 2007 | Saeedi. M | 11 |
| 4 | - | + | + | + | - | - | - | + | 2007 | Yousefi. MR | 12 |
| 3 | + | - | - | + | - | - | - | + | 2007 | Chamani. L | 13 |
| 5 | + | + | + | + | - | - | - | + | 2008 | Mohammadi. P | 14 |
| 6 | + | + | + | + | - | - | + | + | 2008 | Hajghani. H | 15 |
| 5 | - | + | + | + | - | - | + | + | 2008 | Ali Mohammadi. H | 16 |
| 3 | + | - | - | + | - | - | - | + | 2008 | Ziaei kajbaf. T | 17 |
| 5 | + | + | + | + | - | - | - | + | 2009 | Fallahi. Sh | 18 |
| 6 | + | + | + | + | - | - | + | + | 2009 | Arbabi. M | 19 |
| 6 | + | + | + | + | + | - | - | + | 2010 | Jahani Hashemi. H | 20 |
| 4 | - | + | + | + | - | - | - | + | 2010 | Fouladvand. MA | 21 |
| 5 | + | + | + | + | - | - | - | + | 2010 | Fouladvand. MA | 22 |
| 5 | + | + | + | + | - | - | - | + | 2010 | Fouladvand. MA | 23 |
| 5 | + | + | + | + | - | - | - | + | 2011 | Khazaei. HA | 24 |
| 5 | + | + | + | + | - | - | - | + | 2011 | Namayee. MH | 25 |
| 6 | + | + | + | + | - | - | + | + | 2011 | Heydari. A | 26 |
| 6 | + | + | + | + | - | - | + | + | 2012 | Mostafavi. N | 27 |
| 3 | + | - | - | + | - | - | - | + | 2012 | Khazaei. HA | 28 |
| 4 | - | + | + | + | - | - | + | - | 2013 | Maraghi. S | 29 |
| 4 | - | + | + | + | - | - | - | + | 2013 | Rajaii. M | 30 |
| 4 | - | + | + | + | - | - | - | + | 2013 | Ali Asghari. F | 31 |
| 5 | + | + | + | + | - | - | - | + | 2013 | Ghadamgahi. F | 32 |
| 5 | + | + | + | + | - | - | - | + | 2013 | Manouchehri Naeini. K | 33 |
| 3 | + | - | - | + | - | - | - | + | 2013 | Barari-Sawadkohi. R | 34 |
| 6 | + | + | + | + | + | - | - | + | 2013 | Davami. MH | 35 |
| 6 | + | + | + | + | - | - | + | + | 2014 | Kamran. M | 36 |
| 6 | + | + | + | + | - | - | + | + | 2014 | Fallah. E | 37 |
| 6 | + | + | + | + | - | - | + | + | 2015 | Mohammadi.A | 38 |
