# Supplementary material for: Toxoplasmosis seroprevalence in Iranian women and risk factors of the disease: a systematic review and meta-analysis
Source: Trop Med Health. 2017 Apr 12;45:7. doi: 10.1186/s41182-017-0048-7 (PMC5389165; doi:10.1186/s41182-017-0048-7)
Supplement: Supplementary file 2 — Pregnant women group quality assessment table. (DOC 92 kb) [file 41182_2017_48_MOESM2_ESM.doc]

**Pregnant Women Group Quality Assessment Table**

| **Score** | **appropriate statistical methods**  **8** | **valid of survey measures/instruments**  **7** | **reliable of survey measures/instruments**  **6** | **standardized data collection methods**  **5** | **adequate response rate**  **4** | **sample characteristics matching the overall population**  **3** | **representativeness of probability sampling2** | **clear definition of the target population 1** | **Year** | **Author** | **No** |
| --- | --- | --- | --- | --- | --- | --- | --- | --- | --- | --- | --- |
| 5 | + | + | + | + | - | - | - | + | 1994 | Athari. A | 1 |
| 5 | + | + | + | + | - | - | - | + | 1999 | Saffar. MJ | 2 |
| 4 | - | + | + | + | - | - | - | + | 2001 | Talari. SA | 3 |
| 6 | + | + | + | + | - | - | + | + | 2002 | Arbabi. M | 4 |
| 5 | + | + | + | + | - | - | - | + | 2002 | Gharavi. MJ | 5 |
| 5 | + | + | + | + | - | - | - | + | 2002 | Noorbakhsh. S | 6 |
| 5 | + | + | + | + | - | - | - | + | 2003 | Mardani. A | 7 |
| 5 | - | + | + | + | - | - | + | + | 2003 | Talari. SA | 8 |
| 5 | + | + | + | + | - | - | - | + | 2003 | Sotoudeh Jahromi. A | 9 |
| 4 | + | - | - | + | - | - | + | + | 2004 | Sharifi Mood. B | 10 |
| 6 | + | + | + | + | - | - | + | + | 2007 | Manuchehri Naeini. K | 11 |
| 5 | + | + | + | + | - | - | - | + | 2007 | Sohrabi. A | 12 |
| 5 | - | + | + | + | - | - | + | + | 2008 | Cheraghi Pour. K | 13 |
| 5 | + | + | + | + | - | - | - | + | 2008 | Abdi. J | 14 |
| 6 | + | + | + | + | - | - | + | + | 2008 | Fallah. M | 15 |
| 6 | + | + | + | + | - | - | + | + | 2009 | Eskandarian. AA | 16 |
| 3 | + | - | - | + | - | - | - | + | 2010 | Parvizpour. F | 17 |
| 5 | - | + | + | + | - | - | + | + | 2010 | Cheraghi Pour. K | 18 |
| 5 | - | + | + | + | - | - | + | + | 2011 | Ghasemi. A | 19 |
| 5 | + | + | + | + | - | - | - | + | 2012 | Hajsoleimani. F | 20 |
| 5 | - | + | + | + | - | - | + | + | 2012 | Jamshidi Makiani. M | 21 |
| 6 | + | + | + | + | - | - | + | + | 2012 | Dalimi Asl. A | 22 |
| 5 | + | + | + | + | - | - | - | + | 2013 | Siyadat Panah. A | 23 |
| 5 | - | + | + | + | - | - | + | + | 2013 | Akhlaghi. L | 24 |
| 5 | + | + | + | + | - | - | - | + | 2013 | Babaie. J | 25 |
| 5 | - | + | + | + | - | - | + | + | 2013 | Maghsood. AH | 26 |
| 5 | - | + | + | + | - | - | + | + | 2013 | Ebrahimzadeh. A | 27 |
| 4 | - | + | + | + | - | - | - | + | 2013 | Rostami Nejad. M | 28 |
| 5 | - | + | + | + | - | - | + | + | 2013 | Maleki. F | 29 |
| 6 | - | + | + | + | + | - | + | + | 2014 | Vakil. N | 30 |
| 6 | + | + | + | + | - | - | + | + | 2014 | Hoseini. SA | 31 |
| 6 | + | + | + | + | - | - | + | + | 2014 | Mousavi. M | 32 |
| 5 | + | + | + | + | - | - | - | + | 2014 | Kalantari. N | 33 |
| 5 | + | + | + | + | - | - | - | + | 2014 | Sharbatkhori. M | 34 |
| 5 | + | + | + | + | - | - | - | + | 2014 | Yad yad. MJ | 35 |
| 5 | + | + | + | + | - | - | - | + | 2014 | Ghasemloo. H | 36 |
| 5 | + | + | + | + | - | - | - | + | 2014 | Haji Seid Javadi. E | 37 |
| 3 | + | - | - | + | - | - | - | + | 2014 | Elahian Firouz. Z | 38 |
| 6 | + | + | + | + | - | - | + | + | 2014 | Anvari. MH | 39 |
| 5 | - | + | + | + | - | - | + | + | 2014 | Akhlaghi. L | 40 |
| 5 | + | + | + | + | - | - | - | + | 2015 | Rasti. S | 41 |
| 5 | - | + | + | + | - | - | + | + | 2015 | Tabatabaie. F | 42 |
| 5 | + | + | + | + | - | - | - | + | 2015 | Rajaii. M | 43 |
| 5 | + | + | + | + | - | - | - | + | 2015 | Hamidi. M | 44 |
| 5 | + | + | + | + | - | - | - | + | 2016 | Saki.J | 45 |
