# Supplementary figures and images for: Toxoplasmosis seroprevalence in Iranian women and risk factors of the disease: a systematic review and meta-analysis
Source: Trop Med Health. 2017 Apr 12;45:7. doi: 10.1186/s41182-017-0048-7 (PMC5389165; doi:10.1186/s41182-017-0048-7)

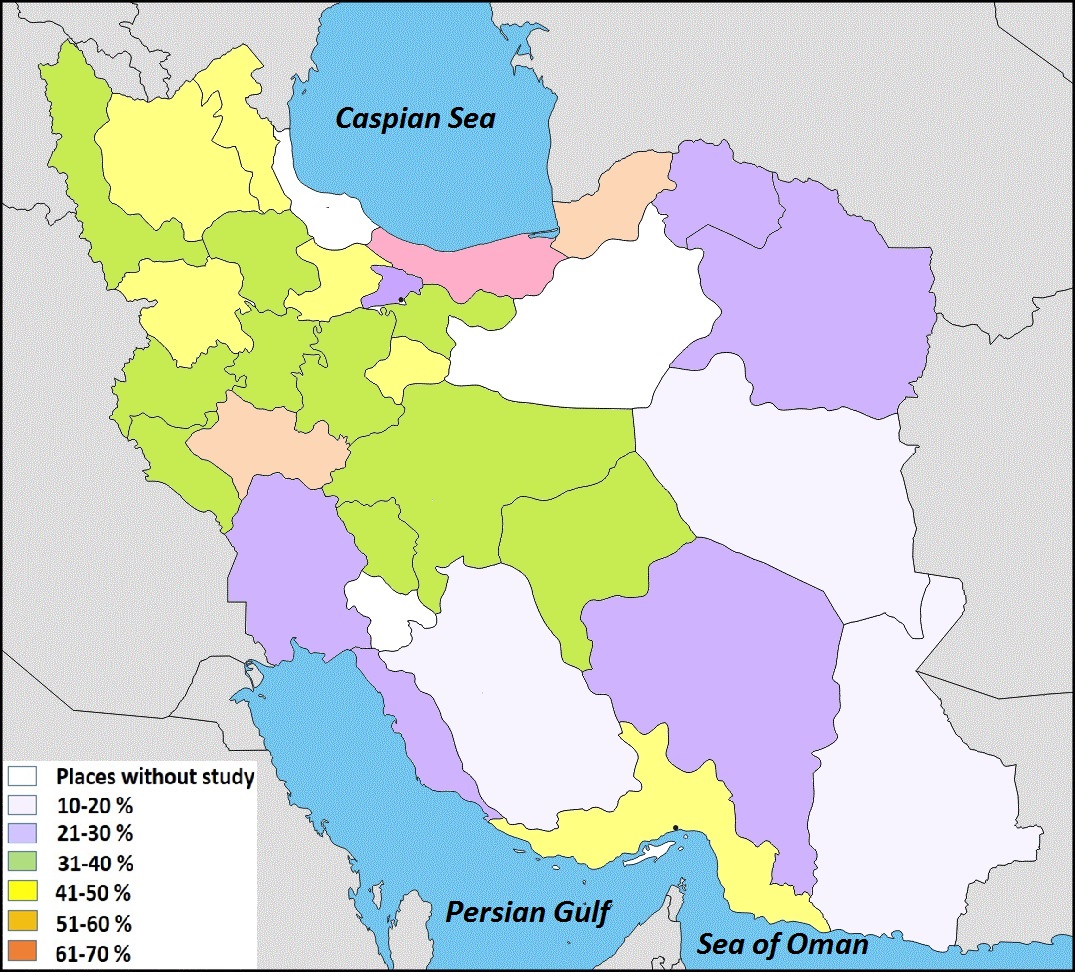

Supplement: Supplementary file 3 — Prevalence rate of Toxoplasma total antibodies in both groups in different provinces of Iran. (JPG 380 kb) [file 41182_2017_48_MOESM3_ESM.jpg]
