# Supplementary material for: Toxoplasmosis seroprevalence in Iranian women and risk factors of the disease: a systematic review and meta-analysis
Source: Trop Med Health. 2017 Apr 12;45:7. doi: 10.1186/s41182-017-0048-7 (PMC5389165; doi:10.1186/s41182-017-0048-7)
Supplement: Supplementary file 4 — Girls and women of childbearing age group methods of included data. (DOCX 16 kb) [file 41182_2017_48_MOESM4_ESM.docx]

| Table S3 Girls and women of childbearing age group methods of included data | | | | | |
| --- | --- | --- | --- | --- | --- |
|  | Kit/Manual | Cut off value  (IgM titer) | Cut off value  (IgG titer) | Method | Authors |
|  | Alphabiol Tech | > 12.5 IU/ml | > 12.5 IU/ml | ELISA | Kamyabi et al., 1999 |
|  | Manual |  | ≥ 1:50 | IFA | Ajami et al., 2001 |
|  | Manual |  | ≥ 1:20 | IFA | Mostafavizadeh et al., 2002 |
|  | NovumDiagnostica |  |  | ELISA | Taravati et al., 2002 |
|  | Manual |  | ≥ 1:20 | IFA | Rabiee et al., 2003 |
|  | Manual |  | ≥ 1:100 | IFA | Mahmoudi et al., 2005 |
|  | RADIM, Italy |  | ≥ 30 IU/ml | ELISA | Hatam et al., 2005 |
|  | Manual |  | ≥ 1:20` | IFA | Fallah et al., 2005 |
|  | Diaplus |  | > 20 IU/ml | ELISA | Rafiei et al., 2005 |
|  | Manual | > 12.5 IU/ml | ≥ 1:20 | IFA | Daryani et al., 2006 |
|  | Kit | >1.1 IU/ml | >1.1 IU/ml | ELISA | Saeedi et al., 2007 |
|  | RADIM, Italy | > 30 IU/ml | > 30 IU/ml | ELISA | Yousefi et al., 2007 |
|  |  |  |  |  | Chamani et al., 2007 |
|  | Biokit, Spain |  | > 10 IU/ml | ELISA | Mohammadi et al., 2008 |
|  | Trinity Biotech |  |  | ELISA | Hajghani et al., 2008 |
|  | ADALTIS, Italy | > 20 IU/ml | > 20 IU/ml | ELISA | Ali Mohammadi et al., 2008 |
|  | Kit |  |  | ELISA | ZiaeiKajbaf et al., 2008 |
|  | Diaplus, USA |  | > 20 IU/ml | ELISA | Fallahi et al., 2009 |
|  | Manual | ≥ 1:20 | ≥ 1:20 | IFA | Arbabi et al., 2009 |
|  | Manual |  | ≥ 1:20 | IFA | Jahani Hashemi et al., 2010 |
|  | Euro Immune, Germany | > 11 IU/ml | > 11 IU/ml | ELISA | Fouladvand et al., 2010 |
|  | Euro Immune, Germany | > 11 IU/ml | > 11 IU/ml | ELISA | Fouladvand et al., 2010 |
|  | Euro Immune, Germany | > 11 IU/ml | > 11 IU/ml | ELISA | Fouladvand et al., 2010 |
|  | DiaSorin, USA | > 12.5 IU/ml |  | ELISA | Khazaie et al., 2011 |
|  | ADALTIS, USA |  | > 20 IU/ml | ELISA | Namayee et al., 2011 |
|  | Viro Immune |  |  | ELISA | Heydari et al., 2011 |
|  | DioPro,Italy |  | > 11 IU/ml | ELISA | Mostafavi et al., 2012 |
|  | Kit |  |  | ELISA | Khazaei et al., 2012 |
|  | Kit | > 1 IU/ml | > 1 IU/ml | ELISA | Maraghi et al., 2013 |
|  | Manual |  | ≥ 1:100 | IFA | Rajaii et al., 2013 |
|  | Trinity Biotech |  | ≥ 1.1 IU/ml | ELISA | Ali Asghari, et al., 2013 |
|  | Euro Immune, Germany | > 11 IU/ml | > 11 IU/ml | ELISA | Ghadamgahi et al., 2013 |
|  | Kit |  |  | ELISA | BarariSawadkohi et al., 2013 |
|  | Dia Pro, Italy | > 11 IU/ml | > 11 IU/ml | ELISA | Davami et al., 2013 |
|  | Dia Pro, Italy |  | > 50 IU/ml | ELISA | ManouchehriNaeini et al., 2014 |
|  | Kit |  | ≥ 1:20 | ELISA | Kamran et al., 2014 |
|  | Kit | > 11 IU/ml | > 11 IU/ml | ELISA | Fallah et al., 2014 |
|  | Manual |  |  | ELISA | Mohammadi et al., 2015 |
