# Supplementary material for: Toxoplasmosis seroprevalence in Iranian women and risk factors of the disease: a systematic review and meta-analysis
Source: Trop Med Health. 2017 Apr 12;45:7. doi: 10.1186/s41182-017-0048-7 (PMC5389165; doi:10.1186/s41182-017-0048-7)
Supplement: Supplementary file 5 — Pregnant women group methods of included data. (DOCX 16 kb) [file 41182_2017_48_MOESM5_ESM.docx]

| Table S2 Pregnant women group methods of included data | | | | |
| --- | --- | --- | --- | --- |
| Kit / Manual | Cut off value  (IgM titer) | Cut off value  (IgG titer) | Method | Authors |
| Manual |  | ≥ 1:50 | IFA | Athari et al.,1994 |
| Raiweil, Italy |  |  | ELISA | Saffar et al., 1999 |
| Manual | ≥ 1:100 | ≥ 1:100 | IFA | Talari et al., 2001 |
| Manual |  | ≥ 1:20 | IFA | Arbabi et al., 2002 |
| Manual |  | ≥ 1:20 | IFA | Gharavi et al., 2002 |
| Clone Systems EIAgen /Biochem  Immuno Systems Italy | ≥ 1.1 | ≥ 1.1 | ELISA | Noorbakhsh et al., 2002 |
| Manual | ≥ 1:20 | ≥ 1:20 | IFA | Talari et al., 2003 |
| Novum Diagnostic |  |  | ELISA | SotoudehJahromi et al., 2003 |
| Equipar |  | ≥ 30 IU/ml | ELISA | Mardani et al., 2003 |
| Manual |  | ≥ 1:20 | IFA | Sharifi Mood et al., 2004 |
| Manual |  | ≥ 1:20 | IFA | Manuchehri et al., 2007 |
| Medac& IBL, Germany /  Trinity Biotech, USA |  |  | ELISA | Sohrabi et al., 2007 |
| Manual |  | ≥ 1:10 | IFA | Abdi et al. ,2008 |
| Manual |  | ≥ 1:20 | IFA | Fallah et al., 2008 |
| Manual |  | ≥ 1:20 | IFA | Eskandarian et al. (2009 |
|  |  |  | ELISA | Parvizpour et al., 2010 |
| Trinity Biotech, Germany | ≥ 1.1 IU/m | ≥ 1.1 IU/ml | ELISA | Cheraghi Pour et al., 2010 |
| Trinity Biotech, USA | ≥ 1.1 IU/ml | ≥ 1.1 IU/ml | ELISA | Cheraghi Pour et al., 2010 |
| Manual |  | ≥ 1:20 | IFA | Ghasemi et al., 2011 |
| Genesis Diagnostic, England |  |  | ELISA | Hajsoleimani et al., 2012 |
| Genesis |  |  | ELISA | JamshidiMakiani et al., 2012 |
| RADIM | OD≥ 30 IU/ml | OD≥ 30 IU/ml | ELISA | DalimiAsl et al., 2012 |
| Dia Pro, Italy | ≥ 11 IU/ml | ≥ 11 IU/ml | ELISA | SiyadatPanah et al., 2013 |
| Manually | >0.343/ ≥1:10 | >0.343 / ≥ 1:10 | ELISA/IFA | Akhlaghi et al., 2013 |
| Euroimmune, Germany | > 1.1 IU/ml | > 1.1 IU/ml | ELISA | Babaie et al., 2013 |
| Euroimmune, Germany | > 1.1 IU/ml | > 1.1 IU/ml | ELISA | Maghsood et al., 2013 |
| PishtazTeb, Iran | ≥ 1.1 IU/ml | ≥ 1.1 IU/ml | ELISA | Ebrahimzadeh et al., 2013 |
| Trinity Biotech, USA | > 1.1 IU/ml | ≥ 1.1 IU/ml | ELISA | RostamiNejad et al., 2013 |
| Kit / Manually | >0.343/ ≥ 1:10 | >0.343/ ≥ 1:10 | ELISA/IFA | Maleki et al., 2013 |
| DIESSE, Italy |  |  | ELISA | Vakil et al., 2014 |
| PishtazTeb, Iran | ≥ 1.1 IU/ml | ≥ 1.1 IU/ml | ELISA | Hoseini et al., 2014 |
| PishtaTeb, Iran | ≥ 1.1 IU/ml | ≥ 1.1 IU/ml | ELISA | Mousavi et al., 2014 |
| Euroimmune, UK | > 11 IU/ml | > 11 IU/ml | ELISA | Kalantari et al., 2014 |
| PishtaTeb, Iran | ≥ 1.1 IU/ml | ≥ 1.1 IU/ml | ELISA | Sharbatkhori et al., 2014 |
| Omega, England |  |  | ELISA | Yadyad et al., 2014 |
| PishtaTeb, Iran | ≥ 1.1 IU/ml | ≥ 1.1 IU/ml | ELISA | Ghasemloo et al. , 2014 |
| Trinity Biotech, USA |  | ≥ 1.1 IU/ml |  | Haji SeidJavadi et al., 2014 |
| Kit |  |  | ELISA | ElahianFirouz et al., 2014 |
| RADIM, Italy |  | ≥ 30 IU/ml | ELISA | Anvari et al., 2014 |
| Dia Pro, Italy | ≥ 11 IU/ml | ≥ 11 IU/ml | ELISA | Akhlaghi et al., 2014 |
| ADALTIS, Italy | ≥ 1:100 | ≥ 1:400 | ELISA | Rasti et al., 2015 |
| Dia Pro, Italy | ≥ 11 IU/ml | ≥ 11 IU/ml | ELISA | Tabatabaie et al., 2015 |
| RADIM, Italy |  | ≥ 30 IU/ml | ELISA | Rajaii et al., 2015 |
| Viro Immune, Germany | ≥ 1:200 | ≥ 1:200 | ELISA | Hamidi et al., 2015 |
| (Biotech Co, England |  |  | ELISA | Saki et al, 2015 |
